# Application Prospect of the SOFA Score and Related Modification Research Progress in Sepsis

**DOI:** 10.3390/jcm12103493

**Published:** 2023-05-16

**Authors:** Xuesong Wang, Zhe Guo, Yan Chai, Ziyi Wang, Haiyan Liao, Ziwen Wang, Zhong Wang

**Affiliations:** 1 School of Clinical Medicine, Tsinghua University, Beijing 100190, China; wxs20@mails.tsinghua.edu.cn (X.W.);; 2 Beijing Tsinghua Changgung Hospital, Tsinghua University, Beijing 100084, China

**Keywords:** sepsis, SOFA score, machine learning

## Abstract

In 2016, the SOFA score was proposed as the main evaluation system for diagnosis in the definition of sepsis 3.0, and the SOFA score has become a new research focus in sepsis. Some people are skeptical about diagnosing sepsis using the SOFA score. Experts and scholars from different regions have proposed different, modified versions of SOFA score to make up for the related problems with the use of the SOFA score in the diagnosis of sepsis. While synthesizing the different improved versions of SOFA proposed by experts and scholars in various regions, this paper also summarizes the relevant definitions of sepsis put forward in recent years to build a clear, improved application framework of SOFA score. In addition, the comparison between machine learning and SOFA scores related to sepsis is described and discussed in the article. Taken together, by summarizing the application of the improved SOFA score proposed in recent years in the related definition of sepsis, we believe that the SOFA score is still an effective means of diagnosing sepsis, but in the process of the continuous refinement and development of sepsis in the future, the SOFA score needs to be further refined and improved to provide more accurate coping strategies for different patient populations or application directions regarding sepsis. Against the big data background, machine learning has immeasurable value and significance, but its future applications should add more humanistic references and assistance.

## 1. Introduction

Sepsis is defined as life-threatening organ dysfunction caused by a dysregulated host response to infection [1]. Sepsis is characterized by high morbidity, high mortality, and a high proportion of medical intervention [2,3]. According to the statistics, the incidence of sepsis has also significantly increased in recent years, with about 300 per 100,000 people diagnosed with sepsis, while 6% of hospitalized patients are diagnosed with sepsis [4,5]. Nearly $24 billion USD was spent on sepsis treatment in 2013, accounting for 13% of U.S. health spending [6,7]. Sepsis has become a major challenge in the field of emergency and critical medical care worldwide [8].

**Figure 1:**
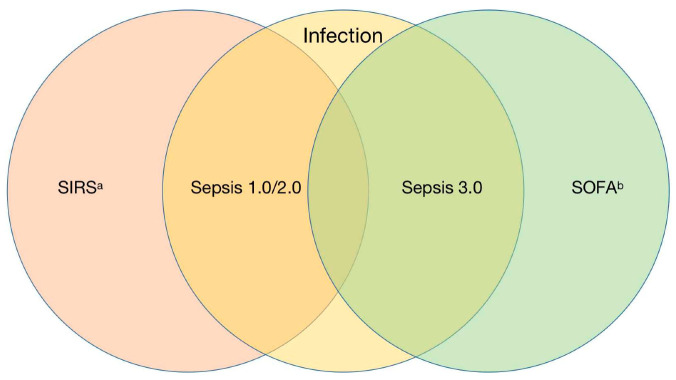
Comparison of new and old diagnosis criteria of sepsis. Abbreviations: SIRS, Systemic Inflammatory Response Syndrome; SOFA, Sequential Organ Failure Assessment. ^a^ Inflammatory response caused by pancreatitis, trauma, burns, etc. ^b^ Sepsis-related SOFA score, life-threatening organ dysfunction caused by dysregulated host response.

The standard nomenclature of sepsis began In 1991 [9]. The term sepsis is a broad term, and there is currently no single clinical standard or imaging or laboratory indicators that can be used to uniquely identify septic patients [10]. Therefore, the definition of sepsis continues to evolve and iterate. According to the third edition of the 2016 definition of sepsis, sepsis is defined as life-threatening organ dysfunction caused by a dysregulated host response to infection [1]. Infection is especially emphasized as the starting point of sepsis, rather than a single pathogen invading the body, and organ failure is regarded as an important sign for the diagnosis of sepsis. This is similar to the definition of severe sepsis in sepsis 1.0/2.0, and the definition of severe sepsis has been removed from sepsis 3.0 [9,11]. The diagnosis scale of sepsis changed from SIRS score to ∆SOFA ≥ 2 (Figure 1). It should be noted that, in the diagnosis of sepsis, although the SIRS score has been replaced by the SOFA score, it still has widespread clinical uses, assisting in determining the degree of infection in patients and predicting the onset of sepsis [12,13]. With the introduction of the third version of the definition of sepsis, people’s understanding of sepsis has become clearer. At the same time, the diagnosis of organ dysfunction is added to the definition of sepsis, indicating that the pathological process of sepsis and the related verification response are more complex [14]. The SOFA score citation plays a very important role in the diagnosis of sepsis (Table 1). Experts believe that sepsis requires a higher level of detection and intervention, and the inclusion of the SOFA score is more convenient for the clinical diagnosis of sepsis while unifying the conceptual differences in the incidence of sepsis, which is conducive to the follow-up development and promotion of sepsis-related research [15].

With the popularization of SOFA score, people have gradually found that the SOFA score has some problems in the diagnosis of sepsis, such as delays in diagnosis, lack of sensitivity, and a complex evaluation [16,17,18]. In view of the above problems, different studies have been carried out regarding the application of the SOFA score to diagnose sepsis. On the one hand, the concept of sepsis is extended, in which many emergency critical field experts, led by Dr. Wang, put forward the concept of preventing and blocking sepsis [19], while some scholars believe that patients who survive sepsis have serious cognitive, mental, and physical disorders, and they put forward the concept of post-sepsis syndrome [20]. Countries have also tried different ways of modifying and optimizing the SOFA score according to their own national conditions. This paper mainly summarizes and analyzes the latest modifications to the SOFA score in various countries and discusses the application prospects and research directions regarding the use of SOFA score in the diagnosis of sepsis.

## 2. The Proposal of the SOFA Score

In the 1980s, people found that severe host response disorder infections can lead to multiple organ failure (MOF), which greatly increases the mortality of critically ill patients [21]. With increases in the understanding of organ function, and to describe the MOF degree of patients as objectively and quantitatively as possible, the European Association of critical Care Medicine formally formulated the SOFA score in Paris in 1994 [22]. The expert group believes that the original intention of establishing the SOFA score was to evaluate organ function damage through a limited number of simple and objective indicators, all of which should be easy to measure in hospitals at all levels and should not exceed the scope of routine testing in the intensive care unit [22]. It is worth mentioning that the expert group agreed that intestinal tissue is the first organ to suffer from functional damage caused by infection, and because intestinal function is too complex and there are no readily available and reliable specific indicators, intestinal function was not included in the evaluation system of SOFA score. This problem has been left until now [23]. The proposal of SOFA score provides a very reliable clinical guidance method to reasonably quantify the degree of organ dysfunction. On this basis, people gradually improve their awareness of multiple organ dysfunction, which provides a favorable reference scheme for clinical diagnosis and treatment. In the continuous development of modern medicine, the SOFA score has always been a recognized and reliable evaluation system in critical medicine [24]. The third edition of sepsis guidelines in 2016 regards SOFA scores as the main evaluation system for the diagnosis of sepsis. SOFA score is no longer limited to applications to the critical care system, but has become a research hot spot in emergency and critical areas and has received more and more attention [25]. With the expansion of the scope of application of SOFA score, it was also found to have many shortcomings, and many related studies on modified SOFA score have been carried out. Different countries and institutions have put forward many new ideas for the SOFA score, but these ideas have not yet been agreed on and are still at the verification stage. We will describe the current modifications to the SOFA score in detail in the following pages.

## 3. Modification of SOFA Score (Related to Sepsis)

**Figure 2:**
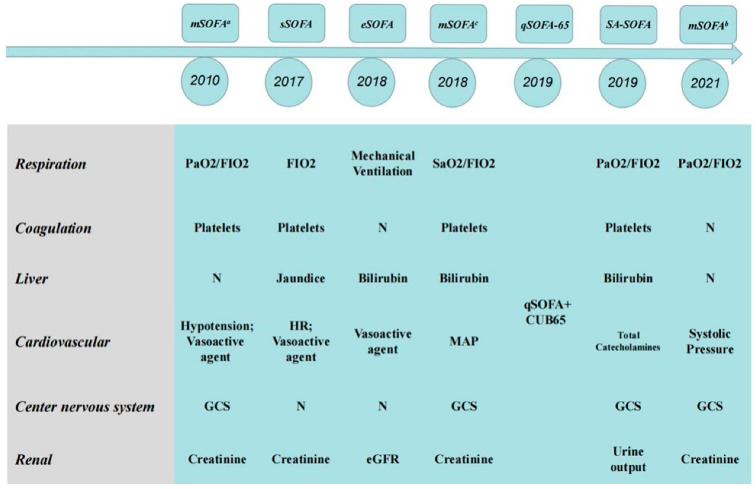
Sepsis-related SOFA modifications. Abbreviations: mSOFA^a^: American Medical Association, 2010; sSOFA: Florida (USA), 2017; eSOFA: The United States Centers for Disease Control and Prevention, 2018; mSOFA^c^: Australian, 2018; qSOFA-65: Republic of Korea, 2019; SA-SOFA: French, 2019; mSOFA^b^: Spain, 2021.

In the previous section, we stated that SOFA score has been a routine method to evaluate the prognosis of patients with multiple organ dysfunction since it was proposed in 1996 [22]. However, there have always been voices challenging the SOFA score; for example, in 2010, the American Medical Association considered it impractical to collect four laboratory parameters in the SOFA score in the event of a massive influx of critically ill patients in an influenza pandemic, natural disasters, or some manmade disasters. Therefore, the laboratory parameters in the SOFA score were cut to a certain extent, and the score was named mSOFAa [26]. However, this modification has not been widely promoted. On the one hand, the purpose of this modification is to deal with sudden public health events and quickly judge the severity of the disease. On the other hand, this scoring system will overlook some critically ill patients who cannot be judged by routine vital signs. After the announcement of new sepsis guidelines in 2016, medical staff in various countries not only adapted and accepted the corresponding new definition of sepsis, but also began to modify the SOFA score for the diagnosis of sepsis. The first station for most patients with sepsis is the emergency room, where the flow of people is large, and monitoring measures are limited. To screen and to identify patients in the emergency room more quickly and to provide timely and positive detection and treatment to patients with risk factors, the SOFA score must urgently be modified. In the nearly six years that have passed since sepsis 3.0 was put forward, countries such as the United States, Australia, Republic of Korea, France, and Spain have successively proposed modified versions of the SOFA score according to national conditions (Figure 2) [27,28,29,30,31]. At the same time, different degrees of improvement tests were carried out according to the low sensitivity of the qSOFA score in the rapid diagnosis of disease. This section will summarize and discuss some of the major SOFA score modifications put forward by countries, as well as the results of the evaluation.

### 3.1. eSOFA Score

eSOFA score is a widespread version of the modified version of SOFA score. From the perspective of monitoring public health events, the SOFA score can predict short-term mortality, but many items in the SOFA score, such as vital signs, vasopressor dose, GCS score, blood gas, FiO_2_ and urine volume, cannot be kept in a structured format in the electronic health record (EHRs). To objectively review and monitor the morbidity and mortality of sepsis, the United States center for Disease Control and Prevention (CDC) released the adult sepsis event surveillance definition, which includes simplified organ dysfunction criteria optimized for electronic health records (eSOFA) [32]. CDC believes that the first consideration for the optimized definition of sepsis for public health surveillance is the reliability and effectiveness of medical institutions, as well as the low burden level of measurement indicators. The timeliness of indicators may not be a limited consideration, as this definition is not used to guide the clinical treatment of individual patients. The main difference between the adult sepsis event standard and sepsis 3.0 is that the eSOFA score can perform automated and consistent sepsis monitoring in hospitals with an EHR system.

In 2019, to compare the similarities and differences between eSOFA score and SOFA score, American experts retrospectively analyzed 942,360 adult cases from 2013 to 2015. The analysis showed that 57,242 (6.1%) met the sepsis 3.0 (SOFA score), while only 41,618 (4.4%) met the adult sepsis event criteria (eSOFA score) [27]. In addition, the eSOFA score was higher than the SOFA score in the identification of hospital mortality (eSOFA, AUROC, 0.774; 95%CI, 0.770–0.779 versus SOFA, AUROC, 0.759; 95%CI, 0.751–0.764. *p* < 0.001). A good overlap can be seen between eSOFA score and SOFA score, and the recognition rate for patients with sepsis is lower, but the mortality is higher. Some problems were also found in the evaluation of eSOFA score. For example, compared with SOFA, the positive predictive value (PPV) could reach 82%, but the sensitivity was only 60%. The main reason for the sensitivity of the eSOFA score is that there is no corresponding standard for hypoxemia and GCS score on the eSOFA score, and only these two aspects can reach the diagnostic standard of ∆SOFA ≥ 2. Therefore, the reasonable expansion of the scope of eSOFA score monitoring could include monitoring sepsis, but it is still necessary to comprehensively consider the impact of various aspects. At present, the definition of eSOFA-related adult sepsis events is also being determined in China [33,34]. As well as comprehensively monitoring sepsis, this is of great significance for guiding the treatment of sepsis and greatly facilitates the exchange and complementation of inter-hospital data.

### 3.2. qSOFA-65 Score and mSOFAb Score

qSOFA does not require laboratory testing and can be evaluated quickly and repeatedly. qSOFA standards can be used to urge clinicians to further investigate patients with organ dysfunction, initiate or upgrade treatment measures as appropriate, and consider transferals to critical care or increasing the rate of test evaluations. It can also promote the consideration of patients that have not been previously identified as infected. In the case of other, more reliable assessments, the qSOFA score is not intended to independently determine sepsis or organ failure [35]. With the continuous development of the research on qSOFA score in recent years, through meta-analysis, the evaluation system of qSOFA was found to be highly specific to the diagnosis of septic organ dysfunction, but its sensitivity is poor, even lower than the SIRS score [36,37,38,39,40,41]. Therefore, it is thought that a more accurate screening scoring system is needed for the early identification of sepsis.

Republic of Korean experts advocate that the age index should be added to the qSOFA score, with an age limit of 65 years old (qSOFA-65) [29]. They believe that an age index is the earliest and most easily available index and would not increase the complexity of the qSOFA score, and age is also an independent risk factor for sepsis, which can be used to evaluate the mortality of patients with sepsis. Therefore, it is feasible to quote age indicators in qSOFA, and a single-center retrospective evaluation found that the sensitivity of the age-based qSOFA-65 score system to sepsis was significantly increased (28%→66%), but the specificity decreased (97%→55%) [29]. The 65-year-old threshold determined by qSOFA-65 score is not suitable for all countries, and populations from each country show obvious diversity, so it is necessary to pay attention to the characteristics of the population while referring to the evaluation system. In addition, the impact of age indicators on qSOFA still needs to be evaluated by large, multi-center samples.

Based on the previously noted problem, Spanish experts chose to establish a new, pre-hospital first evaluation system, mSOFAb [31]. In this score, all laboratory indicators can be obtained through point-of-care testing (POCT), while other scores are immediately evaluated by first-aid personnel. A prospective, multi-center study found that patients with more than 6 points should prioritize emergency treatment, with a higher early mortality rate, while those with less than 6 points had a mortality rate of less than 10%. The key point of this score is to put forward the important role of POCT pre-hospital and in the emergency room. Whether the SOFA score or the other scoring system are used to diagnose severe diseases, we strive for a continuous balance between the simplicity and accuracy of prognosis based on the objective reliability of evaluation indicators. POCT has the obvious advantages of rapid inspection. In recent years, with the extensive development of various green channels, POCT has become an indispensable important link. In the modified quantitative evaluation system for the rapid diagnosis of sepsis, the function of POCT may need to be widely studied and discussed in the future.

### 3.3. SA-SOFA Score

French experts have discussed sepsis-related SOFA scores mainly to assess the long-term prognosis of patients. They believed that the critical values of indicators and variables considering the SOFA score are mainly generated by the consensus of experts and lack of favorable, evidence-based medicinal support. Another shortcoming of the SOFA score is that the recent ‘Sepsis surviving campaigns’ progress in critical care has limited its performance in predicting mortality, which deeply changed the early care of septic patients; for example, dopamine is now seldom used, whereas norepinephrine is the reference drug for septic shock [42]. All these lead to a decrease in the specificity of SOFA scores in predicting mortality in patients with sepsis. Therefore, French experts designed and verified a new, simplified and more accurate SOFA score (SA-SOFA score) [30]. A retrospective study of 1436 patients found that SA-SOFA score was superior to SOFA score in predicting 28-day mortality. The proposal of SA-SOFA score solves an important defect in the SOFA score, its poor accuracy in predicting long-term mortality, but it also has some limitations, such as the high requirements for testing. Therefore, the role of the early diagnosis of sepsis is limited. In addition, there may be only one stage of severe circulatory decompensation in the use of catecholamines; although the specificity in predicting mortality is very high, the corresponding sensitivity is poor.

The principles and background proposed by the SA-SOFA are in line with the framework of SOFA score updates, but the evaluation indicators still need to be further discussed and supported by evidence-based medicine, as well as multi-center prospective experiments.

### 3.4. Other Modified Versions of the SOFA Score

In addition to the above modification of SOFA scores, some countries and regions have also put forward different suggestions for the sepsis-related modification of SOFA score; for example, Florida (USA) proposed a sSOFA score [43], while Australian experts proposed mSOFAc [28]. These scores simplify the SOFA score or change the index to varying degrees, but these scores have not been evaluated by large, multi-center samples. Florida proposed the sSOFA score after preliminary evaluation; its sensitivity and specificity are slightly lower than that of the SOFA score, but the evaluation system is simpler and more feasible [43]. Australian experts proposed mSOFAc, based on systolic blood pressure, fluid resuscitation response, and vasoactive drug use, to reflect the circulatory system, as well as SpO_2_ to reflect the respiratory system; the other system evaluation is the same as the SOFA score. Through the evaluation of infected emergency patients in many hospitals, the negative predictive value of mSOFAc score was 97.9% (95%CI, 0.939–0.996) [28]. As mentioned above, the modified SOFA scores should be different with different applications, and no scoring system can meet all the requirements. On the one hand, the purpose of these modified versions of the SOFA score is not clear; and, on the other hand, if the sample size is too small, the credibility of retrospective research is insufficient, so its prospects need to be further verified.

## 4. Application of Modified SOFA Score to Sepsis-Related Concepts

As shown in the modifications to SOFA score introduced in the previous section, there is no scoring system that can meet everyone’s clinical needs; different clinical needs will produce different modified versions of the SOFA score. The clinical treatment of sepsis is very complicated in the absence of a gold standard. The third edition of the definition of sepsis aims to describe sepsis more clearly; infection and organ dysfunction are regarded as the only two indicators for the diagnosis of sepsis [44]. These two indicators meet the standard for the classification and description of sepsis. However, the diagnosis of sepsis faces a diversity of clinical problems, such as the earlier screening of patients suspected of sepsis, standardization of the treatment window and treatment after sepsis is diagnosed, as well as how to prevent sepsis patients from being admitted to hospital again. These problems cannot be solved by the definition of sepsis alone. In view of this, experts and scholars in the field of emergency and critical care in various countries also discussed the definition of sepsis and provided suggestions or launched initiatives. Among them, the most famous is the Surviving Sepsis Campaign, and a 1 h bundle treatment was suggested [45,46]. These new concepts or definitions of sepsis provide guidance for patients with complex sepsis and provide clear modification directions and guidelines for SOFA scores. A more accurate range of application will result in a more realistic SOFA score. In this chapter, we summarize and discuss several extended definitions of sepsis and the application prospects of the discussed SOFA score.

### 4.1. Surviving Sepsis Campaign (SSC)

In 2002, the SSC was put forward by the European Society of Intensive Care Medicine (ESICM) and Society of Critical Care Medicine (SCCM) [47]. Since the first edition of the SSC guidelines was launched in 2004, it has been updated to version 2021, which has become an important international standard for the treatment of sepsis [48]. The 2021 version of the SSC guidelines contains six parts: screening and early treatment, infection, hemodynamic management, mechanical ventilation, supportive treatment, long-term outcomes, and care goals, with a total of 93 items and 99 recommendations [49]. It is worth noting that the evaluation systems used in sepsis screening in the guidelines include the SIRS score, qSOFA score, SOFA score, NEWS score, and MEWS score, which differ in the evaluation of sepsis and have poor predictive value. These guidelines also make it clear that qSOFA score alone is not recommended as a screening tool for sepsis or septic shock. qSOFA score, as a tool for the rapid diagnosis and screening of sepsis, as specified in the definition of sepsis 3.0, has received objections. The guidelines point out that qSOFA score has high specificity in the diagnosis of sepsis, but its sensitivity is poor, which will cause missed diagnoses in patients with sepsis; therefore, qSOFA score alone is not recommended as a screening tool for sepsis [50]. This suggests that the 2021 version of the SSC guidelines is pessimistic about the existing tools for early screening for sepsis. In addition, another important point in the guidelines is the addition of long-term outcomes and care goals, and 20 recommendations were provided. These included treatment objectives, palliative care, a peer support team, treatment handover, sepsis knowledge education, shared decision-making, discharge planning, and out-of-hospital follow-up. The SSC guidelines invite sepsis patients and their relatives from different countries and backgrounds to participate in the development of this part. This shows that the treatment of sepsis should not be limited to hospital treatment, but should be measured by a longer-term goal.

In the 2021 edition of the SSC guidelines, the evaluation system used to evaluate organ function damage and mortality in septic patients is still the SOFA score, and there is no discussion on the modified SOFA score. However, this unilaterally negates the value of qSOFA score as an independent scoring system to identify early sepsis patients. On the one hand, this shows that the SOFA score is still applicable to the diagnosis and prediction of sepsis; and, on the other hand, it also reflects that the modification of SOFA score is a long process, which may be based on the great progress made at the medical testing level. As an internationally recognized guide for the treatment of sepsis, the SSC guidelines are worthy of reference and should be learned by clinicians, but in the treatment of septic patients, we should pay more attention to individual differences and avoid rigid treatment plans.

### 4.2. Prevention and Intervention of Sepsis

Most experts involved in the 2021 version of the SSC guidelines are involved in the field of intensive care, and their focus is still on the treatment of sepsis organ function and the later stages of sepsis treatment. For the same disease, the focus of different disciplines is not the same, and there are differences in their understanding of the nature of the disease and their ideas regarding diagnosis and treatment [51]. Experts from the field of emergency medicine in China initiated and put forward the project of ‘Preventing Sepsis Campaign in China, PSCC’ [19]. The theoretical basis of the PSCC project is that the process from infection to sepsis is continuous and interventionable, moving from local and mild inflammation to severe inflammation. Once systemic organ injury occurs, it is difficult to recover. In the consensus of emergency experts on the prevention and blocking of sepsis in China, three scoring systems that can be used to screen for sepsis are recommended, including qSOFA score ≥ 2, SOFA score = 1, and NEWS score = 4–6 [17,52,53]. One of the three scores meets the criteria and, on the premise of clear clinical evidence of infection, the patient can be assessed as having suspected sepsis [19].

In this chapter, we will not elaborate too much on the clinical treatment of suspected sepsis or the early prevention of sepsis. Rather, we will only discuss the scoring system provided in the PSCC consensus to judge patients with suspected sepsis. First, the three evaluation systems selected in the PSCC consensus did not provide high-quality statistical support to the data, similar to the SOFA score, which mainly depends on clinical expert advice. Although the SOFA score has withstood various tests, indicating that the lack of evidence-based medicine at the early stage cannot negate the proposal of a new idea, it is still an indispensable link to improve the theory [54]. Secondly, the consensus recommends ∆SOFA = 1 as one of the criteria, and there is some controversy regarding the critical value of the SOFA score. On the one hand, the specificity of the judgement of ∆SOFA = 1 in the cognitive range is not high. On the other hand, some experts believe that the main application of the SOFA score should be in the evaluation of multiple organ dysfunction, and there are many scoring systems that can replace the SOFA score in the evaluation of single organ function. It is doubtful whether this can play an effective early warning role in patients with sepsis; many prospective studies will be discussed in the follow-up. Finally, the connection between the three scoring systems given in the consensus is independent; suspected sepsis can be identified by satisfying a particular scoring system. This recommendation still needs to be further measured and discussed. For example, it has been recognized that the qSOFA score is less sensitive, and the 2021 version of the SSC Guidelines no longer recommends qSOFA as a means of evaluation for the rapid diagnosis of sepsis [55]. In addition, the NEWS score is too tedious compared to the qSOFA score. Therefore, more specific suggestions should be made regarding the prioritization of application scenarios for the three scoring systems.

The original intention of the PSCC project must be correct. The treatment of sepsis should not be limited to the intensive sepsis care unit, but should move to a more cutting-edge emergency field. The earlier the intervention and treatment, the better the prognosis of the septic patients [56]. Although the SSC Guidelines allowed for early screening and treatment in the first part of each version, they still focus on how to save organ function, and they do not pay enough attention to early diagnosis and treatment [57]. In the above chapter on the modifications made to the SOFA score, we mentioned the modification results of the qSOFA score from Republic of Korea and Spain, respectively. Republic of Korea added age factors on the basis of qSOFA score, while Spain put forward new testing items and indicators on the basis of POCT and achieved better results than qSOFA score in the preliminary verification, providing us with new ideas and directions regarding the modification of qSOFA score [29,31]. After evaluating and modifying the existing evaluation system, can we further put forward a new evaluation system, which is suitable for the medical environment of various countries? A more reasonable screening of patients with early suspected sepsis still needs more work and evaluation, and further information should be gathered during the accumulation process.

### 4.3. Post-Sepsis Syndrome

**Figure 3:**
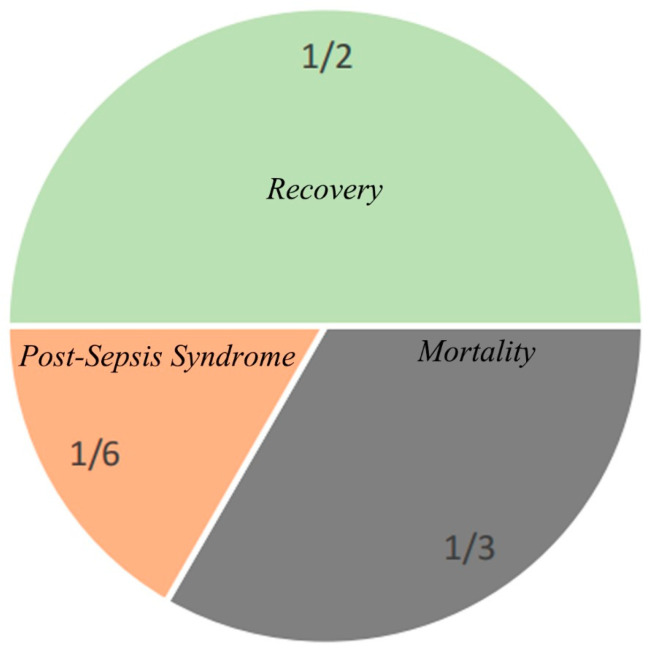
Patient outcomes following sepsis.

With the standardized treatment of sepsis, about 14 million patients diagnosed with sepsis survive and leave hospital each year [58]; about half of these patients fully or nearly fully recover, and 1/3 of them die during this period. A total of 1/6 of these patients have one or more serious and lasting complications (Figure 3). In view of the various complications that occur after sepsis, some people put forward the concept of post-septic syndrome [20]. Post-sepsis Syndrome refers to the subsequent cognitive, mental, physical, and other physiological dysfunction of severe sepsis. The concept is similar to the long-term outcome and care goals presented in the 2021 version of the SSC Guidelines. Post-sepsis syndrome not only takes up a lot of medical resources, but also causes great trouble to the patients’ quality-of-life. According to 20 suggestions provided in the long-term outcome and care goals section of the SSC Guidelines, the current situation of post-sepsis syndrome can be improved, but this needs to be based on a larger health care system, which is not realistic for some developing countries or countries with relatively poor medical resources. If septic patients with a high probability of post-sepsis syndrome can be screened and evaluated during hospitalization, targeted treatment plans and out-of-hospital follow-up can save medical resources [59]. In the previous chapter, we introduced the idea that SOFA score is not effective in predicting long-term mortality in septic patients. For this reason, experts from France proposed a version of the SA-SOFA score to improve the poor long-term prognosis of septic patients evaluated by the SOFA score [30]. In addition, age and underlying disease level should also be independent factors for predicting post-sepsis syndrome. Learning from the SOFA score evaluation system to establish an evaluation system that is suitable for predicting post-sepsis syndrome may be the main means of predicting and evaluating post-sepsis syndrome in the future.

## 5. Comparison of Machine Learning and SOFA Score in the Early Screening and Diagnosis of Sepsis

Compared with the traditional modification of SOFA score under different sepsis-related concepts, machine learning has made rapid breakthroughs in the field of medicine in recent years [60]. The principle of machine learning is to continuously learn and obtain valuable conclusions while analyzing the inductive data, which are based on the popularity and promotion of clinical big data [61]. With the establishment and promotion of the database in the field of critical medicine, machine learning has achieved a series of research results in the early prediction of sepsis, accurate treatment of sepsis, population classification of sepsis, prediction of the outcome of patients with sepsis, etc. [62,63,64,65,66]. The value of machine learning in the diagnosis and treatment of sepsis has received increasing attention and recognition [67]. The theme of the PhysioNet/CinC Challenge 2019 is the ‘early prediction of sepsis based on clinical data in ICU’. This competition attracted more than 100 teams from around the world, and the organizers provided a total of more than 60,000 ICU patients, with a total duration of more than 2 million hours [68,69]. The success of this event has caused the application of machine learning in sepsis to reach a new level. This chapter mainly describes the current machine learning results on the early prediction and diagnosis of sepsis compared with the traditional SOFA/qSOFA score, to further identify areas with application potential for the early identification and diagnosis of sepsis.

The application direction of machine learning is the same as in traditional score systems, such as SOFA score. Different models can be used to predict the different stages and prognosis of the disease [70]. The relationship with the SOFA score is opening a new track in the same field and will hopefully overtake this in the short term. Machine learning methods are mainly divided into decision tree, support vector machine, random forest bell, gradient boosting, neural network, and deep learning [71,72]. In 2016, Deasutels built an insight prediction model based on the MIMIC-III database by using heart rate, respiratory rate, body temperature, systolic blood pressure, pulse pressure, blood sample saturation, GCS score, and age data [73]. The AUROC for predicting sepsis was 0.880. In 2018, Nemati constructed an artificial intelligence sepsis expert (AISE) early warning model based on 65 kinds of real-time data from 27,527 critically ill adult patients, which could provide a warning regarding the occurrence of sepsis [74]. In 2019, Scherpf et al. developed a recursive neural network (RNN) model which can make better use of the time-dependent pattern of data, and the UAROC also reached 0.810 [75]. In 2020, Burdick used the gradient enhancement method to develop an early warning model based on the routine physiological indexes of 270,438 emergency adult patients, and the AUROC was 0.827 [76]. At the PhysioNet/CinC Challenge 2019, Yang et al., from Southeast University of China, extracted 168 clinical characteristic variables from 34,285 critically ill adult patients using eXtreme Gradient Boosting (XGBoost) and trained an explainable AI sepsis predictor (EASP) [68]. This model can help predict the risk of sepsis in advance, and its prediction accuracy, sensitivity, and specificity are 0.85, 0.90, and 0.74, respectively. This was verified on the test databases from different hospital systems.

The advantage of machine learning is obvious; even under limited conditions, machine learning still shows better predictability. Compared with the traditional SOFA score and other scoring systems, machine learning can divide different indicators and critical values in more detail and present the prediction results more directly. At present, in view of the continuous expansion of clinical sepsis index data, it is impossible to rely on manual screening and identification; we can only find differences in different permutations and combinations to meet the corresponding clinical needs. However, depending on the machine learning model, we only need to give the demand to obtain an effective conclusion. Although the conclusion is not unique, it can be further screened artificially [77]. If relying on machine learning to diagnose sepsis, we do not need to worry about false-positive results because false-positive results also have a high reference value [78]. However, under the condition of excessive dependence on machine learning, the occurrence of false-negative results may be very important to patients’ prognosis. Although the accuracy of each machine learning model is high, the object of comparison is still a traditional scoring system. We think that this kind of comparison is insufficient. The data magnitude and evaluation indicators used by the two evaluation methods are not at the same level. Since, once the auxiliary diagnosis and treatment of machine learning enters clinical practice, it is bound to produce high dependence, and the goal is to completely avoid the occurrence of false-negative events. This is in line with the current research direction of other high-tech industries, such as autopilot technology. We have no way to install only 99% of the successful autopilot modes on every car; this will inevitably lead to directly related casualties. In the future, the research and development direction of machine learning should first be more precise, which is consistent with the SOFA score modification, and we can only obtain the best answer in a limited range. At present, the machine learning model for predicting sepsis is still based on the big data of ICU [79]. In fact, the data set in the field of emergency and pre-hospital first-aid should be used to predict sepsis. The second aim is to improve accuracy as much as possible. The establishment of trust requires thousands of experiments, but the collapse of trust only needs to occur one time.

The continuous development of machine learning in the medical field not only brings more optimized solutions to the clinic, but also provides clinical decision-makers with more choices and challenges [77,80]. Clinical treatment is not a single digital exchange, but also includes social and personal factors, which cannot be solved by machine learning at present. Although machine learning is in its infancy, it has a bright future. We cannot unilaterally deny the advantages of emerging technologies just because of their shortcomings. Every technological innovation will face such challenges; how to develop and mature technology is not the most important, while how to deal with the challenges brought by technological development most needs to be determined.

## 6. Conclusions

Currently, most SOFA score modifications are mainly focused on different application systems or the national conditions of different countries [81]. These improvements cannot deny the SOFA score. The current controversy over SOFA scores revolves around the definition of sepsis 3.0 [82]. As there is no gold standard for the diagnosis of sepsis, the SOFA score used to define sepsis is fully in line with people’s need for a definition of sepsis, and the problem should be the clinical diagnosis and treatment of sepsis. In addition, the SOFA score does not distinguish sepsis caused by different pathogens. There are significant differences in the treatment of sepsis caused by different pathogens, and the prognosis of patients is also not similar [83]. In addition, the patient’s age, living environment, and other existing diseases are all closely related to the diagnosis and progression of sepsis. The importance of these indicators and their necessity as diagnostic screening indicators should receive further discussion in the future.

Compared with the traditional SOFA scoring and other evaluation systems, the different models produced by machine learning have a higher accuracy and could be used to discover more aspects that people have not paid attention to before, such as finding new sepsis population types and evaluating the efficacy of the targeted treatment of sepsis. However, machine learning does not have any explanation, and there is no way to form an effective means of communication between doctors and patients. Once clinicians become over-dependent on the results of machine learning, which leads to irreparable medical accidents, this becomes impossible for the current health care system to solve. Machine learning provides us with more choices and ways to find problems, but the final choice and decision must be people-oriented.

Taken together, we believe that the rapid development of sepsis has become a trend, and a single SOFA score cannot meet all the needs of sepsis research. It is necessary to make reasonable SOFA modifications after clarifying the concepts and research needs related to sepsis. However, as an important evaluation system for the cognitive and auxiliary diagnosis of sepsis, the SOFA score is still reliable at present.

## Figures and Tables

**Table 1:** Sequential (Sepsis-Related) Organ Failure Assessment Score.

	Score				
System	0	1	2	3	4
**Respiration**					
PaO_2_/FIO_2_, mmHg (kPa)	≥400 (53.3)	<400 (53.3)	<300 (40)	<200 (26.7) with respiratory support	<100 (13.3) with respiratory support
**Coagulation**					
Platelets, ×10^3^/μL	≥150	<150	<100	<50	<20
**Liver**					
Bilirubin, mg/dL (μmol/L)	<1.2 (20)	1.2–1.9 (20–32)	2.0–5.9 (33–101)	6.0–11.9 (102–204)	>12.0 (204)
Cardiovascular	MAP ≥ 70 mmHg	MAP < 70 mmHg	Dopamine < 5 or dobutamine (any dose) ^a^	Dopamine 5.1–15 or epinephrine ≤ 0.1 or norepinephrine ≤ 0.1 ^a^	Dopamine > 15 or epinephrine > 0.1 or norepinephrine > 0.1 ^a^
**Central nervous system**					
Glasgow Coma Scale score	15	13–14	10–12	6–9	<6
**Renal**					
Creatinine, mg/dL (μmol/L)	<1.2 (110)	1.2–1.9 (110–170)	2.0–3.4 (171–299)	3.5–4.9 (300–440)	>5.0 (440)
Urine output, mL/d				<500	<200

Abbreviations: FIO_2_, fraction of inspired oxygen; MAP, mean arterial pressure; PaO_2_, partial pressure of oxygen. ^a^ Catecholamine doses are given as μg/kg/min for at least 1 h.

## Data Availability

No applicable.
